# Supplementary material for: Electrochemical System for Field Control of Hg2+ Concentration in Wastewater Samples
Source: Sensors (Basel). 2023 Jan 17;23(3):1084. doi: 10.3390/s23031084 (PMC9919054; doi:10.3390/s23031084)
Supplement: Supplementary file 1 [file sensors-23-01084-s001.zip › sensors-2146937-supplementary.pdf]

SUPPLEMENTARY INFORMATION

Electrochemical system for field control of Hg^{2+} concentration in wastewater samples

Anda-Gabriela Tenea#[1,2], Cristina Dinu#[1], George-Octavian Buica*[3], Gabriela-Geanina Vasile*[1]

[1]National Research and Development Institute for Industrial Ecology ECOIND, 57-73 Drumul Podul Dambovitei Str., 060652, Bucharest, Romania, anda.tenea@incdecoind.ro (A.G.T.); cristina.dinu@incdecoind.ro (C.D.); gabriela.vasile@incdecoind.ro (G.-G.V.);

[2] Science Faculty, Chemistry Department, University of Craiova, 107i Bucharest Street, 200478 Craiova, Romania

[3] University Politehnica of Bucharest, Faculty of Chemical Engineering and Biotechnologies, 1-7 Polizu Str., 011061, Bucharest, Romania, george.buica@upb.ro (G.-O.B.)

*Correspondence: george.buica@upb.ro (G.-O.B.); gabriela.vasile@incdecoind.ro (G.-G.V.).
#Authors with equal contribution

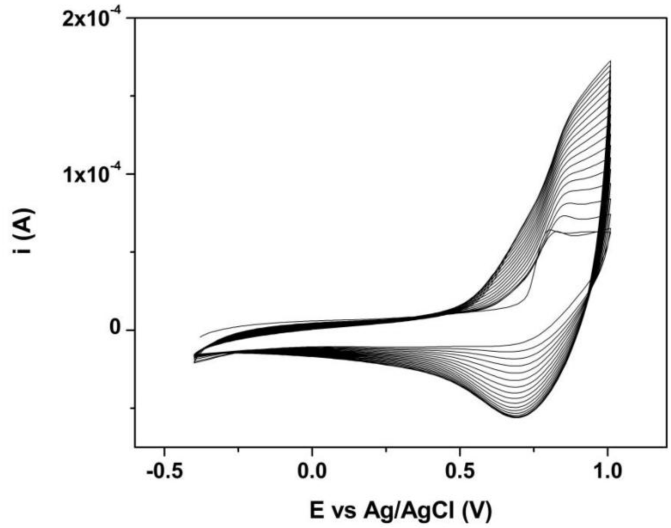

Figure S1. Succesive cyclic voltamograms recorded in 1.5mM **L** in 0.1M TBAP, acetonitrile solution at carbon SPE (0.1 V/s).

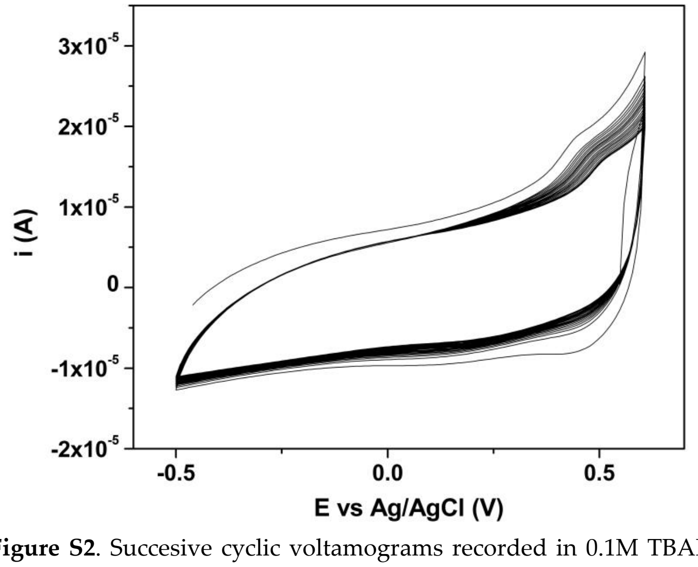

Figure S2. Succesive cyclic voltamograms recorded in 0.1M TBAP, acetonitrile solution at SPE-poly**L** modified electrode (0.1 V/s).

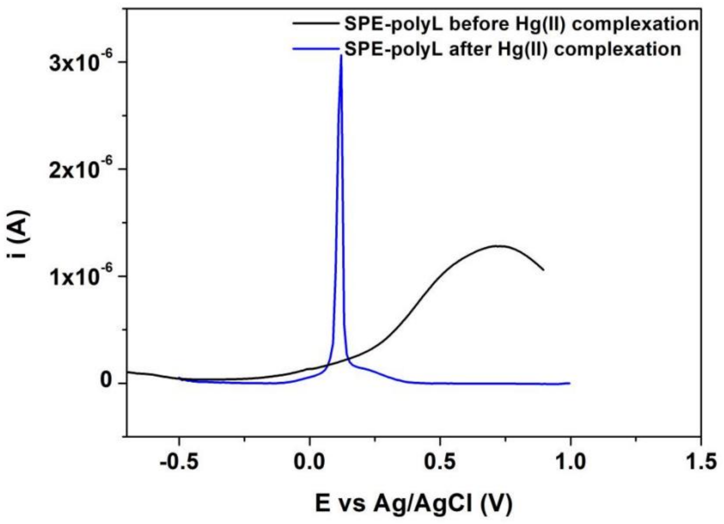

Figure S3. DPV stripping curves recorded on SPE electrode modified with poly**L** before and after mercury complexation; the accumulation was performed in acetate buffer at pH 3.0 containing 10^{-7} M Hg(II) for a time of 20 min; and the reduction was performed at -1.3 V for 15 seconds.

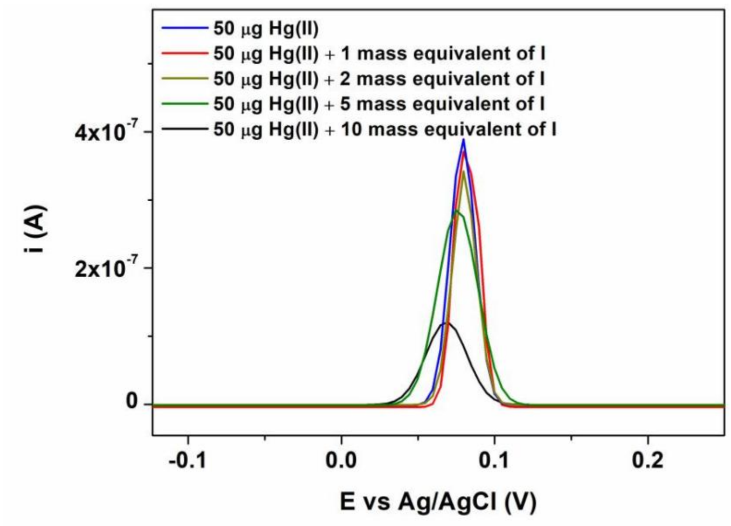

Figure S4. Effect of interfering cations (I: (Zn(II), Cd(II), Pb(II), Ni(II), Co(III), and Cu(II)) on 50 μg Hg(II) detection after open-circuit accumulation of 20 min in acetate buffer (pH = 3). Each interfering ions is at the same specified mass equivalent vs. Hg(II) ions concentration.
